# Isolation, purification and characterization of novel antimicrobial compound 7-methoxy-2,2-dimethyl-4-octa-4′,6′-dienyl-2*H*-napthalene-1-one from *Penicillium* sp. and its cytotoxicity studies

**DOI:** 10.1186/s13568-015-0120-9

**Published:** 2015-07-04

**Authors:** Harpreet Kaur, Jemimah Gesare Onsare, Vishal Sharma, Daljit Singh Arora

**Affiliations:** Department of Microbiology, Microbial Technology Laboratory, Guru Nanak Dev University, Amritsar, 143005 India; Department of Pharmaceutical Sciences, Guru Nanak Dev University, Amritsar, 143005 India

**Keywords:** Antimicobial, Fungi, *Penicillium*, Purification, Cytotoxicity

## Abstract

Fungus isolated from soil has been evaluated for its antimicrobial activity which showed broad spectrum antimicrobial activity against all the pathogenic microorganisms used. Optimization was done by response surface methodology (RSM) to further optimize the medium which could further enhance the antimicrobial activity by 1.1–1.9 folds. Column chromatography was used to isolate the active compound which was characterized to be by various spectroscopic techniques such NMR, IR and LCMS and it was found to be apparently novel compound **7-methoxy-2,2-dimethyl-4-octa-4′,6′-dienyl-2*H*-napthalene-1-one**. MIC of the active compound ranged from (0.5–15 µg/mL which was found to be comparable with the standard antibiotics. Viable cell count studies of the active compound showed it to be bactericidal in nature. Further, the compound when tested for its biosafety was found neither to be cytotoxic nor mutagenic. Cytotoxicity studies of the compound on cancer cell lines showed a valuable cytotoxic potential against all tested human cancer cell lines. Further, the compound induces apoptosis in lung cancer (A549) cells reveled by increase the distribution of nuclear DNA in Sub G1 phase as observed in flow cytometry. The study demonstrated that an apparently novel compound isolated from *Penicillium* sp. seems to be a stable and potent antimicrobial.

**Electronic supplementary material:**

The online version of this article (doi:10.1186/s13568-015-0120-9) contains supplementary material, which is available to authorized users.

## Introduction

Microorganisms have been used as a source for the production of variety of bioactive metabolites. These include bacteria, algae, fungi, actinomycetes etc. Numbers of bioactive metabolites have been isolated from these natural sources. The discovery and development of antibiotics was one of the most significant advances in the medicine in the 20th century. Till now many antibiotics have been commercialized to fight against various diseases. The bacterial resistance is spreading throughout the world, revealing the steadily decreasing potencies of relevant antibiotics (Gould 2008) thus necessitating the discovery of novel compounds or modification of already existing antimicrobial stock. Now days, multiple drug-resistant microorganisms such as methicillin-resistant *Staphylococcus aureus* (MRSA), vancomycin-resistant *S. aureus* (VRSA), vancomycin-resistant *Enterococci* (VRE), have increased around the world (Gould 2008). Extended spectrum *β*-lactamases (ESBLs) producing *Escherichia coli*, *Klebsiella pneumoniae* and *Pseudomonas aeruginosa*, are becoming resistant to almost every available antibiotic (Livermore 2009). Soil holds an enormous biodiversity that can be screened for antibiotic production. Because of huge expenditure on synthetic molecules with effective antimicrobial properties, natural products are still worth promise (Newman and Cragg 2007).

Filamentous fungi are widely distributed in the environment. The wonder drug directed the interest of the researchers towards natural resources having different biological activities. Since then, a number of compounds with antibiotic activity have been isolated from different fungi such as, *Penicillium janczewskii, Penicilliumcanescens* (Kozlovskii et al. 1997), *Penicillium sclerotiorium, Penicillium janthinellum, Penicillium citrinum* (Takahashi et al. 2008), *Myrothecium cinctum* (Kobayashi et al. 2004) and *Aspergillus fumigatus* (Furtado et al. 2005), etc. Fungi are historically important sources of secondary metabolites and they continue to provide new chemical entities with novel biological activities (Baker and Alvi 2004). They provide a rich source of compounds for therapeutic applications including antibacterial (Takahashi et al. 2008), antifungal (Nicoletti et al. 2007), antiviral (Nishihara et al. 2000), immunosuppressant and cholesterol-lowering agents (Kwon et al. 2002). Number of fungi showing differ rent biological activities have been listed in the literature; still a lot remains untapped from diverse soil habitats. Screening of novel strains is bringing about microorganisms, not yet assayed for their antibacterial activity that can yield innovative molecules or useful templates for development of new antibiotics (Takahashi et al. 2008). There is a wide variety of fungi used in the production of these compounds including *Penicillium* spp. which are also exploited to produce bioactive molecules such as antibiotics, enzymes, and organic acids. A large number of fungal extracts and or extracellular products have been found to possess antimicrobial activity, mainly from the filamentous fungi *Penicillium* (Petit et al. 2009). Many *Penicillium* spp. have been screened for bioprocessing since the discovery of penicillin, still many of the species and strains remain untapped which may be useful for various pharmaceutical purposes. Taking cognizance of wide biological importance of *Penicillium* spp., the present study was designed to isolate and screen the fungi from soil collected from different areas of Punjab (30° 4′ N, 75° 5′ E), India. One such promising isolate, *Penicillium* sp. showing antimicrobial activity was selected for isolation, purification and characterization of the compound.

## Materials and methods

### Test organisms

The reference strains of bacteria and yeasts were obtained from Microbial Type Culture Collection (MTCC), Institute of Microbial Technology (IMTECH), Chandigarh, India and the clinical isolate methicillin resistant *Staphylococcus aureus* (MRSA) was obtained from Post graduate Institute of Medical Education and Research (PGIMER), Chandigarh, India. Reference strains included Gram positive bacteria: *Enterococcus faecalis* (MTCC-439), *S. aureus* (MTCC-740), *Staphylococcusepidermidis* (MTCC-435), Gram negative bacteria: *E. coli* (MTCC-119), *K. pneumoniae* 1 (MTCC-109), *K. pneumoniae* 2 (MTCC-530), *P. aeruginosa* (MTCC-741), *Salmonella typhimurium* 1 (MTCC-98), *S. typhimurium 2* (MTCC-1251), *Shigella flexneri* (MTCC-1457) and two yeast strains: *Candida albicans* (MTCC-227), and *Candida tropicalis* (MTCC-230).

### Fungal isolation and extract preparation

Fungus was isolated as described earlier (Kaur et al. 2014) using yeast extract glucose agar (YGA) medium. Fifty millilitre YPDS medium (g/L) with pH-6, taken in 250 mL flasks, was inoculated with four fungal discs (8 mm) of 6–7 days old culture grown on YGA. After 7 days of incubation at 25°C as stationary culture, the contents were filtered through Whatman filter paper no. 1 and the filtrate obtained was used for assaying its antimicrobial potential.

### Antimicrobial screening

#### Inoculum preparation

A loopful of bacterial and yeast colonies were respectively inoculated into 5 mL of nutrient broth and yeast malt medium and incubated at 37 and 25°C, respectively for 4 h. The turbidity of actively growing microbial suspension was adjusted to match the turbidity standard of 0.5 Mc Farland units (Kaur and Arora 2009). The inoculum thus prepared was used to assay their sensitivity to fungal extract by agar well diffusion assay (Bauer et al. 1996).

### Statistical optimization of the medium

On the basis of our previous results (Arora and Kaur 2011) obtained from screening of different carbon and nitrogen sources through one-factor-at-a-time classical method; starch, dextrose and yeast extract were taken as independent variables for further optimization by response surface methodology (RSM) using Box–Behnken design (Arora and Sharma 2011; Box and Behnken 1960). Each variable was studied at three levels (−1, 0, +1); for starch and dextrose these were 0.5, 1.25 and 2%, while for yeast extract it was 0.4, 1.2 and 2%. The experimental design included 17 flasks with five replicates having all the three variables at their central coded values. The mathematical relationship of response G (for each parameter) and independent variable X (X_1_, dextrose; X_2_, Starch; X_3_, Yeast extract) was calculated by the following quadratic model equation.

$$ {\text{G}} = \beta_{0} + \, \beta_{ 1} {\text{X}}_{ 1} + \, \beta_{ 2} {\text{X}}_{ 2} + \, \beta_{ 3} {\text{X}}_{ 3} + \, \beta_{ 1 1} {\text{X}}_{ 1}^{ 2} + \, \beta_{ 2 2} {\text{X}}_{ 2}^{ 2} + \, \beta_{ 3 3} {\text{X}}_{ 3}^{ 2} + \, \beta_{ 1 2} {\text{X}}_{ 1} {\text{X}}_{ 2} + \, \beta_{ 1 3} {\text{X}}_{ 1} {\text{X}}_{ 3} + \, \beta_{ 2 3} {\text{X}}_{ 2} {\text{X}}_{ 3} $$

where, G is the predicted response; β0 is intercepted; β1, β2 and β3 are linear coefficients; β11, β22 and β33 are squared coefficients and β12, β13 and β23 are interaction coefficients. MINITAΒ statistical software was used to obtain optimal working conditions and generate response surface graphs.

### Isolation and purification of bioactive compound

For the extraction and purification of active group/component from *Pencillium* sp., 3 L of the culture broth was extracted with equal volume of butanol (1:1). The organic layer was separated and treated with Na_2_SO_4_ and then evaporated to dryness in vacuum and the resulting solids 4.5 g were subjected to column chromatography using silica gel (100–200 mesh size, column 18 mm × 300 mm; Hi-media) packed and pre-equilibrated with hexane. The column was first eluted with equilibration solvent i.e. hexane (two bed volumes) followed by linear gradients of hexane: chloroform (100:0, 90:10, 80:20, 70:30, 60:40, 50:50, 40:60, 30:70, 20:80, 10:90, 0:100) at a flow rate of 1 mL/min followed by linear gradients of hexane: ethyl acetate (100:0, 90:10, 80:20, 70:30, 60:40, 50:50, 40:60, 30:70, 20:80, 10:90, 0:100). Different fractions of 20 mL each were collected and after concentration were subjected to agar disc diffusion assay and thin layer chromatography. Hexane: chloroform (8:2) was used as screening system to develop the chromatograms which were observed under UV light (254 and 365 nm) and in iodine chamber. Fractions which showed similar TLC pattern were pooled, concentrated and again loaded to silica gel (100–200 mesh size), column (10 mm × 300 mm) and pre-equilibrated with hexane (two bed volumes). The column after elution with hexane was eluted with linear gradients of Hexane: Chloroform (as above) and fraction size reduced to 5 mL. The collected fractions were tested for antimicrobial activity and thin layer chromatography. The single bands with similar Rf value were pooled and again checked for antimicrobial activity against *S. aureus* and *C. albicans* with disc diffusion assay. These pooled single bands were then loaded to HPLC and were further subjected to various spectroscopic analyses.

### HPLC analysis

The purified active fractions as obtained above were further subjected to high pressure liquid chromatography (HPLC) using Dionex P680 HPLC. Acetonitrile (75% aqueous solution) was used as mobile phase at a flow rate 0.3 mL/min and injection volume was 20 µL at a column temperature of 25°C. The detections were monitored at different wavelengths (225, 250, 275 and 300 nm).

### Spectroscopic analysis

The purified fraction from column chromatography was further subjected to various spectroscopic analyses for the characterization of the compound. Bruker AC-400 FT (400 MHz) spectrometer was used to record 1H and 13C nuclear magnetic resonance (NMR) spectra. Chemical shifts (δ) are reported as downfield displacements from trimethylsilane (TMS) used as internal standard and coupling constants are reported in Hz. Infrared (IR) spectra was recorded on Varian 660-IR Fourier Transform Infra-Red Spectrometer in KBr pellets coated with chloroform film. A mass spectrum by EI and ESI methods was recorded on Bruker microTOF using ESI-TOF method.

### Minimum inhibitory concentration (MIC)

Minimum inhibitory concentration of the butanolic extract as well as for the purified compound was worked out by agar dilution method (Kaur and Arora 2009). A stock solution of butanolic extract (25 mg/mL) and for purified compound (10 mg/mL) concentration was prepared and incorporated into Mueller–Hinton agar medium for bacteria and yeast malt extract medium for yeast. The final concentrations of the compound in the medium containing plates ranged from (0.1–20 mg/mL) for butanolic extract and (0.5–15 µg/mL) for pure compound. The experiment was performed in duplicate along with standard drugs (gentamicin 0.059 mg/mL and amphotericin B 0.75 mg/mL).

### Time kill assay

A stock solution 25 mg/mL of butanolic extract of the fungus and for the purified compound stock solution of (10 mg/mL) was prepared and the time kill assay for butanolic extract and the purified compound was performed by viable cell count method (VCC) (Kaur and Arora 2009).

### Post antibiotic studies

The PAE of butanolic extract and the compound was studied as described by Raja et al. (2011). The extract was mixed with each test organism (in equal volume) containing approximately 1 × 10^5^ cfu/mL suspended in suitable broth medium. After 2 h exposure the drug activity was stopped by placing 1:1,000 dilution of microbial suspension in a broth drug free pre-warmed suitable double strength broth. CFU sampling was done at an interval of 1 h until visual cloudiness was noted. The PAE was calculated as follows PAE = T − C, where T represents the time required for the count in the test culture to increase 1log 10 cfu/mL above the count observed immediately after drug removal and C represents the time required for the count of the untreated control tubes to increase by 1log 10 cfu/mL.

### Biosafety evaluation

#### Ames mutagenicity testing

Butanolic extract and the purified compound were subjected to both spot and plate incorporation methods of Ames test to evaluate their mutagenicity (Maron and Ames 1983). This *Salmonella* reverse mutation test is based on histidine dependence and mutations in *S. typhimurium* (MTCC-1251, IMTECH, Chandigarh, India). In case of plate method, 0.1 mL of the bacterial inoculum was added which was adjusted according to its MIC value and 0.1 mL of butanolic extract (25 mg/mL) as well as purified compound (10 mg/mL) to 5 mL of top agar containing 0.25 mL of 0.5 mM histidine–biotin mixture (1:1 ratio). The contents were mixed and poured onto glucose minimal agar plates immediately. Similarly, the same protocol was followed for spot method except that butanolic extract and the purified compound were impregnated on to disc which was placed in the centre of the plate. Sodium azide (5 μL of 17.2 mg/mL) was used as a positive control while DMSO was used as negative control. The plates were prepared in duplicate and incubated at 37°C for 48 h. The number of visible revertant colonies was counted. The mutagenic potential of the extracts was determined on the basis of number of colonies as compared to positive control.

### Cellular toxicity testing using MTT assay

The cellular toxicity of butanolic extract and the purified compound was determined by MTT (3-[(4, 5-dimethylthiazol-2-yl)-2, 5-diphenyl] tetrazolium bromide) assay (Ciapetti et al. 1993). Ten millilitre sheep blood was taken into injection syringe containing 3 mL Alsever’s solution (anticoagulant) and transferred to sterile centrifuge tubes. The blood was centrifuged at 1,600×*g* at room temperature (25 ± 3°C) for 20 min to separate the plasma from the cells. The supernatant was discarded and centrifuged again adding 6 mL phosphate buffer saline (PBS). The blood cells were washed thrice with PBS by centrifugation and the pellet was resuspended in 6 mL of PBS. Various dilutions of these cells using PBS were prepared and counted with the help of a haemocytometer under a light microscope so as to obtain cells equivalent to 1 × 10^5^ cells. The following formula was used to determine the required number of cells: number of cells/mL = number of cells counted in 25 squares × dilution factor × 10^4^. The cell suspension thus prepared was dispensed into Elisa plates (100 µL/well) and incubated at 37°C for overnight. The supernatant was removed carefully and 200 µL of the extract and the purified compound was added and incubated further for 24 h. Supernatant was removed again and added 20 µL MTT solutions (5 mg/mL) to each well and incubated further for 3 h at 37°C on orbital shaker at 60 rpm. After incubation, the supernatant was removed without disturbing the cells and 50 µL DMSO was added to each well to dissolve the formazan crystals. The absorbance was measured at 590 nm using an automated microplate reader (Biorad 680-XR, Japan). The wells with untreated cells served as control.

### Cytotoxic activity

The sulforhodamine B (SRB) assay is a colorimetric assay used for cytotoxic screening to assess cell growth inhibition (Skehan et al. 1990). Cells are cultured in a 96 well tissue culture plate and the cell growth which depends upon the rate of multiplication is measured indirectly by the intensity of the color of the dye which is directly proportional to the number of cells present. The human cancer cell lines (procured from N.C.I., Frederick, USA) were grown in tissue culture flasks in complete growth medium (RPMI-1640 with 2 mM glutamine, pH 7.4, supplemented with 10% fetal calf serum, 100 µg/mL streptomycin and 100 units/mL penicillin) in a carbon dioxide incubator (37°C, 5% CO_2_, 90% RH). The cells at sub-confluent stage were harvested from the flask by treatment with trypsin [0.05% in PBS (pH 7.4) containing 0.02% EDTA]. Cells with viability of more than 98% as determined by trypan blue exclusion were used. For determination of cytotoxicity the cell suspension of 1 × 10^5^ cells/mL was prepared in complete growth medium. Stock solution of the purified compound was prepared in DMSO and it was serially diluted with complete growth medium containing 50 µg/mL of gentamicin to obtain working test solutions of required concentrations. In vitro cytotoxicity against five human cancer cell lines of different tissues was determined using 96-well tissue culture plates. The 100 µL of cell suspension was added to each well of the 96-well tissue culture plate. The cells were allowed to grow in carbon dioxide incubator (37°C, 5% CO_2_, 90% RH) for 24 h. Test materials in complete growth medium (100 µL) were added after 24 h of incubation to the wells containing cell suspension. The plates were further incubated for 48 h in a carbon dioxide incubator. The cell growth was stopped by gently layering trichloroacetic acid (50%, 50 µL) on top of the medium in all the wells. The plates were incubated at 4°C for 1 h to fix the cells attached to the bottom of the wells. The liquid of all the wells was gently pipette out and discarded. The plates were washed five times with distilled water to remove trichloroacetic acid, growth medium, low molecular weight metabolites, serum proteins etc. The plates were stained with sulforhodamine B dye [0.4% (w/v) in 1% acetic acid (v/v), 100 µL] for 30 min. The plates were washed five times with 1% acetic acid and then air-dried (Skehan et al. 1990). The adsorbed dye was dissolved in Tris–HCl buffer (100 µL, 0.01 M, pH 10.4) and plates were gently stirred for 10 min on a mechanical stirrer. The optical density (OD) was recorded on ELISA reader at 540 nm. The cell growth was determined by subtracting mean OD value of respective blank from the mean OD value of experimental set. Considering the growth in the absence of any test material as 100%, percent growth inhibition was calculated. IC_50_ values were determined by non-linear regression analysis using Graph Pad Software (2236 Avenida de l Playa La Jolla, CA 92037, USA).

### Flow cytometry analysis of nuclear DNA

Analysis of nuclear DNA by flow cytometry is interesting in fundamental research and has broadly contributed to improved knowledge on cell DNA content and their distribution in the various phases of cell cycle. DNA amount in cells is often the single parameter measured for cell cycle studies by flow cytometry. In order to obtain a linear relationship between cellular fluorescence intensity and DNA amount, analyses are performed with fluorescent molecules that bind specifically and stoichiometrically to DNA. Some dyes possess an intercalative binding mode such as propidium iodide etc., whereas others such as Hoechst 33342, DAPI etc. possess an affinity for DNA A-T rich region. To analyze the nuclear DNA content, lung cancer cells (2 × 10^6^ cells/mL/well) were treated with purified compound for 24 h at concentrations 10 and 20 µM, and washed twice with ice-cold PBS, harvested, fixed in cold 70% ethanol in PBS and stored at −20°C for 30 min. After fixation, the cells were incubated with RNase A (0.1 mg/mL) at 37°C for 30 min, stained with propidium iodide (50 µg/mL) for 30 min and then measured for nuclear DNA content using BD-LSR Flowcytometer (Becton–Dickinson, USA) equipped with electronic doublet discrimination capability using blue (488 nm) excitation from argon laser.

### Statistical analysis

The values have been presented as mean of three independent experiments. Single factor analysis of variance (ANOVA) was used to calculate the significant difference.

## Results

### Antimicrobial screening

The isolated fungus was identified as *Penicillium* sp. close to *P. citrinum* group by National Fungal Culture Collection of India (NFCCI), Agharkar Research Institute, Pune, India (Accession No. NFCCI 2555) Extracellular culture broth of the selected fungus. It showed antimicrobial activity against two Gram positive bacteria: *S. aureus* (34 mm) *S. epidermidis* (36 mm) and MRSA (17 mm), one Gram negative bacteria: *K. pneumoniae* 1 (17 mm) and one yeast *C. albicans* (18 mm),while rest of the organisms were found to be resistant to extracellular culture broth. Further, to enhance the antimicrobial activity, RSM experiment was designed using the carbon and nitrogen sources.

Box–Behnken design for statistical optimization of carbon and nitrogen sources for antimicrobial activity of *Penicillium* sp.

On the basis of results obtained from screening of different carbon and nitrogen sources through one-factor-at-a-time classical method; these were optimized by RSM using Box–Behnken design of experiments

- *S. aureus* (G_1_)
  $$ \begin{aligned}   32.4 &  + 6.87{\text{ X}}_{1}  + 1.25{\text{ X}}_{2}  - 2.1{\text{ X}}_{3}  - 3.0{\text{ X}}_{1}^{2}  - 5.32{\text{ X}}_{2}^{2}  \\     &  - 1.57{\text{ X}}_{3}^{2}  + 0.5{\text{ X}}_{1} {\text{X}}_{2}  - 2.75{\text{ X}}_{1} {\text{X}}_{3}  + 2.0{\text{ X}}_{2} {\text{X}}_{3}  \\  \end{aligned}  $$

**Figure 1:**
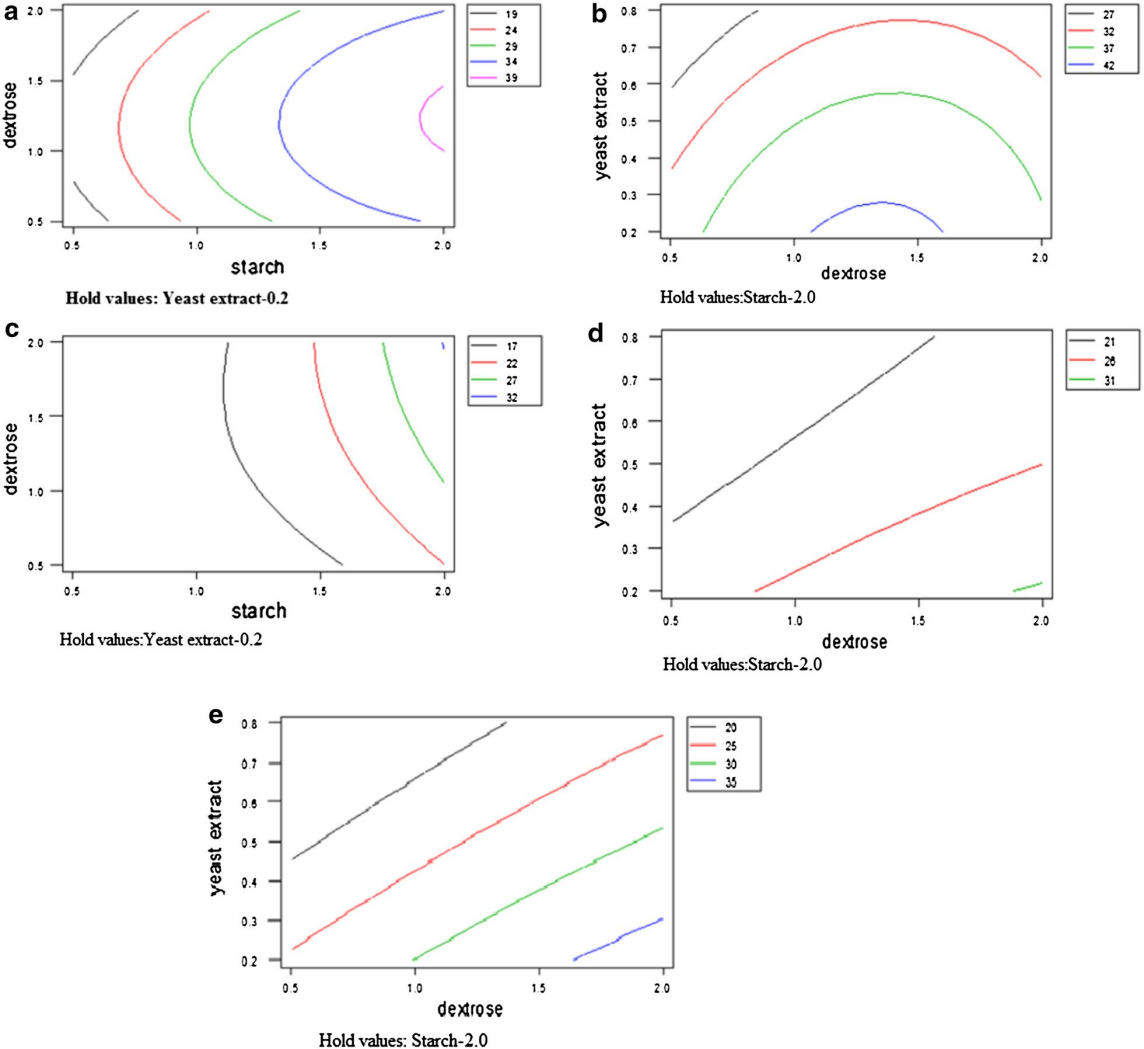
**a** Contour *plot* of *Staphylococcus aureus.* **b** Contour *plot* of *Staphylococcus epidermidis.* **c** Contour *plot* of *Klebsiella pneumoniae* 1. **d** Contour *plot* of *MRSA.* **e** Contour *plot* of *C. albicans*.

Linear effect of starch and squared effect of dextrose was highly significant with P value ≤0.001. Similarly linear effect of yeast extract and squared effect of starch was significant with P value ≤0.05 and R^2^ value of 94.4%. The response surface graph showed the highest activity at 2% starch, 1–1.5% dextrose, with yeast extract 0.2% (Figure 1a).

- *S. epidermidis* (G_2_)
  $$ \begin{aligned} 3 2. 2&+ 7. 2 5 {\text{ X}}_{ 1} + 0. 5 {\text{ X}}_{ 2} - 2. 5 {\text{ X}}_{ 3} - 1. 1 {\text{ X}}_{ 1}^{ 2} - 6. 6 {\text{ X}}_{ 2}^{ 2} \\&- 1. 6 {\text{ X}}_{ 3}^{ 2} + 2 {\text{ X}}_{ 1} {\text{X}}_{ 2} - 3. 5 {\text{ X}}_{ 1} {\text{X}}_{ 3} + 1.0{\text{ X}}_{ 2} {\text{X}}_{ 3} \end{aligned} $$

Linear effect of starch and squared effect of dextrose was highly significant with P value ≤0.001. Similarly linear effect of yeast extract was found to be significant with P value ≤0.05. Interactive effect of starch and yeast extract was also found to be significant P value ≤0.05 and R^2^ value observed was 92.7%. The response surface graph showed the highest activity at 1–1.5% dextrose, starch 2% and yeast extract 0.2% (Figure 1b).

- *K.pneumoniae* 1 (G_3_)
  $$ \begin{aligned}6. 2&+ 4. 1 {\text{ X}}_{ 1} + 1. 8 7 {\text{ X}}_{ 2} - 1. 2 5 {\text{ X}}_{ 3} + 3. 1 5 {\text{ X}}_{ 1}^{ 2} - 1. 3 5 {\text{ X}}_{ 2}^{ 2} \\&+ 0. 4 {\text{ X}}_{ 3}^{ 2} + { 3}.0{\text{ X}}_{ 1} {\text{X}}_{ 2} - 3. 2 5 {\text{ X}}_{ 1} {\text{X}}_{ 3} - 0. 2 5 {\text{ X}}_{ 2} {\text{X}}_{ 3}\end{aligned} $$

Linear effect of starch was highly significant with P value ≤0.001. Linear effect of dextrose and squared effect of starch was also significant with P value ≤0.05. Interactive effect of starch and yeast extract, starch and dextrose was significant with P value ≤0.05. R^2^ value observed was 91.3%. The response surface graph showed the highest activity at starch 2%, dextrose 1.5–2% and yeast extract 0.2% (Figure 1c).

- *MRSA* (G_5_)
  $$ \begin{aligned}1 6. 4&+ 3. 2 5 {\text{ X}}_{ 1} + 1. 7 5 {\text{ X}}_{ 2} - 1 {\text{ X}}_{ 3} + 3. 4 {\text{ X}}_{ 1}^{ 2} - 0. 5 7 {\text{ X}}_{ 2}^{ 2} \\&+ 0. 9 2 {\text{ X}}_{ 3}^{ 2} + 1. 7 5 {\text{ X}}_{ 1} {\text{X}}_{ 2} - 3. 2 5 {\text{ X}}_{ 1} {\text{X}}_{ 3} - 0. 2 5 {\text{ X}}_{ 2} {\text{X}}_{ 3}\end{aligned} $$

Linear effect of starch was highly significant with P value ≤0.005. Squared effect of starch and interactive effect starch and yeast extract was significant with P value ≤0.05 and R^2^ value of 87.1%. The response surface graph showed the highest antimicrobial activity at dextrose 1.5–2%, starch 2% and yeast extract 0.2% (Figure 1d).

- *C. albicans*
  $$ \begin{aligned}1 7. 4&+ 4. 1 2 {\text{ X}}_{ 1} + 2. 1 2 {\text{ X}}_{ 2} - 2.0{\text{ X}}_{ 3} + 3. 9 2 {\text{ X}}_{ 1}^{ 2} - 0. 5 7 {\text{ X}}_{ 2}^{ 2} \\&+ 0. 1 7 {\text{ X}}_{ 3}^{ 2} + 3. 7 5 {\text{ X}}_{ 1} {\text{X}}_{ 2} - 4. 5 {\text{ X}}_{ 1} {\text{X}}_{ 3} - 0{\text{ X}}_{ 2} {\text{X}}_{ 3} \end{aligned}$$

Linear effect of starch was highly significant with P value ≤0.005. Linear effect of dextrose and squared effect of starch was significant with P value ≤0.05. Interactive effect of starch and dextrose; starch and yeast extract was significant with P value ≤0.05 and R^2^ value of 90.8%. The response surface graph showed the highest antimicrobial activity at dextrose 1.5–2%, starch 2% and yeast extract 0.2% (Figure 1e).

### Validation of results

Thus from the overall assessment 2% starch, dextrose 1–2%, yeast extract 0.2% and 1% peptone in YPDS medium may be regarded as the optimized conditions for antimicrobial activity. The F value and R^2^ value showed that the model correlated well with measured data and was statistically significant. To confirm the adequacy of the model the verification experiments using optimum medium composition as described above were carried out in triplicates. The antimicrobial activity (Table 1) was enhanced by 1.1 folds in *S. aureus* and *S. epidermidis*. However, an increase of 1.8 fold was observed for *K. pneumoniae*1 and MRSA while in *C. albicans*, it increased by 1.9 folds.

**Table 1:** Result of Box–Benhken design experiment for antimicrobial activity of *Penicillium* sp

Std.	Dextrose	Starch	Yeast extract	*S. aureus*	*S. epidermidis*	*K. pneumoniae*	*C. albicans*	*MRSA*
	(g/100 mL)			Zone of inhibition (mm)				
1	0.5	0.5	0.5	15	17	14	17	15
2	0.5	2	0.5	27	28	16	17	18
3	2	0.5	0.5	20	17	14	17	17
4	2	2	0.5	34	36	28	32	27
5	1.25	0.5	0.2	20	20	15	16	17
6	1.25	2	0.2	40	41	30	34	30
7	1.25	0.5	0.8	21	25	16	18	18
8	1.25	2	0.8	30	32	18	18	18
9	0.5	1.25	0.2	30	30	14	17	15
10	2	1.25	0.2	25	26	16	18	17
11	0.5	1.25	0.8	22	20	15	16	17
12	2	1.25	0.8	25	20	16	17	18
13	1.25	1.25	0.5	33	34	16	17	16
14	1.25	1.25	0.5	30	28	17	17	17
15	1.25	1.25	0.5	32	33	16	18	17
16	1.25	1.25	0.5	35	33	16	18	16
17	1.25	1.25	0.5	32	33	16	17	16

### Isolation and purification of bioactive compound

For the extraction and purification of active group/component from *Penicillium* sp., three liters of the culture broth was extracted with equal volume of butanol (1:1). All the fractions (120) obtained from column chromatography were pooled according to similar pattern of chromatogram on TLC plates. Three sets of fractions (A, B and C) were obtained from column chromatography having similar R_f_ value and antimicrobial activity against various pathogenic bacteria and yeast strains such as *S. aureus*, *S. epidermidis*, *K. pneumoniae* 1, MRSA, *C. albicans*, *S. typhimurium* 1, *S. typhimurium* 2, *Sh. flexneri* and *E. coli*. The second set (B) of pooled fraction showed a single spot on TLC with R_f_ value (0.65 cm). It was further subjected to HPLC analysis to determine the purity of active compound which showed single peak at retention time 8.643 min. However, Set A and C also showed antimicrobial activity but had some impurities. First set (A) showed antimicrobial activity with zone of inhibition ranging from 20 to 23 mm followed by second set (B) which showed zone of inhibition ranging from 17 to 20 mm against various microbial strains. As the second set (B) showed single spot on TLC, it was pursued for further spectroscopic analysis. The compound responsible for antimicrobial activity was characterized by various spectroscopic techniques such as IR, ^1^H & ^13^C NMR and mass. **Colour of compound: Yellowish brown: IR** (KBr, CHCl_3_): λ_max_ = 2,940, 2,915, 2,876, 1,666, 1,651, 1,535, 1,454, 1,373, 1,284, 1,161, 1,107, 1,072, 968, 756 cm^−1^; ^**1**^**H NMR** (400 MHz, CDCl_3_): δ 9.35 (s, 1H, C8-H), 8.26–8.12 (d, 1H, *J* = 8.0 Hz C5-H), 7.61 (d, 1H, *J* = 8.0 Hz, C6-H), 7.39–7.20 (m, 2H, C4′-H & C6′-H), 6.91–6.84 (m, 3H, C5′-H, C7′-H & C3-H), 4.37 (s, 3H, OCH_3_), 2.04 (t, 2H, *J* = 5.2 Hz, C1′-H), 1.92–1.87 (m, 2H, C2′-H), 1.40–1.24 (m, 2H, C3′-H), 1.11 (s, 6H, 2 × CH_3_); ^**13**^**C NMR** (100 MHz, CDCl_3_): δ 170.2 (C=O), 169.1 (olefinic CH), 166.4 (arom. q), 163.7 (olefinic CH), 160.1 (arom. q), 147.9 (C4), 125.6 (olefinic CH), 124.5 (olefinic CH), 121.9 (olefinic CH), 118.8 (arom. CH), 116.2 (arom. CH), 67.0 (OCH_3_), 57.2 (q), 45.8 (CH2), 44.5 (CH_2_), 27.8 (CH_2_), 21.9 (CH_3_), 19.1 (CH_3_); **HR-MS** (TOF, ESI): *m*/*z*: calculated for C_21_H_26_O_2_ + [Na]: 333.1803; found: 333.1766 [M + Na]^+^ (Additional file 1: Figure S1).

### Minimum inhibitory concentration (MIC)

Equal volume of the culture broth from *Penicillium* sp. was extracted with equal volume of butanol in separating funnel. The organic phase was separated and the solvent was then evaporated to dryness under vacuum and the residue obtained was reconstituted in dimethyl sulfoxide (DMSO). Minimum inhibitory concentration was worked out for butanolic extract of the fungus as well as for the purified compound by agar dilution method (Table 2). Butanolic extract showed significant antimicrobial activity with MIC 0.1 mg/mL against *S. aureus,* and *K. pneumoniae* 1, followed by MIC 0.2 mg/mL for *S. epidermidis* and *C. albicans*, against MRSA, 0.7 mg/mL, *S. typhimurium* 2 and *P. aeruginosa* 1 mg/mL. The butanolic extract also showed good inhibitory activity against *Sh. flexneri* and *E. faecalis* with MIC 5 mg/mL, followed by MIC value of 10 mg/mL and 20 mg/mL against *S. typhimurium* 1 and *K. pneumoniae* 2, respectively. MIC of purified compound against *C. albicans* and *K. pneumoniae* 1 with 1 µg/mL as compared to standard antibiotics, amphotericin B (99 µg/mL) and gentamicin (1 µg/mL). It was followed by MIC 2 µg/mL against *S. aureus* and *S. epidermidis* whereas against MRSA 5 µg/mL and *S. typhimurium* showed highest MIC (10 µg/mL).

**Table 2:** Comparison of MIC of butanolic extract and pure compound with two standard antibiotics

Microorganisms	MIC butanolic extract	MIC of compound	MIC of gentamicin	MIC of amphotericin B
*S. aureus*	0.1^c^	2^b^	1^a^	ND
*S. epidermidis*	0.2^c^	2^b^	1^a^	ND
*K. penumoniae 1*	0.1^c^	1^b^	0.19^a^	ND
*C. albicans*	0.2^b^	1^a^	ND	ND
*MRSA*	0.5^c^	5^b^	0.19^a^	99^d^
*P. aeruginosa*	1^b^	ND	10^a^	ND
*S. typhimurium 2*	0.7^c^	5^b^	2^a^	ND
*Sh. flexneri*	5^b^	ND	2^a^	ND
*E. faecalis*	5^b^	ND	10^a^	ND
*E. coli*	10^b^	ND	1^a^	ND
*S. typhimurium 1*	15^b^	ND	1^a^	ND
*K. penumoniae 2*	20^b^	ND	1^a^	ND

The values represents mean of three values, different lower case letters (a–c) show statistical significant (P ≤ 0.05) difference between columns.

### Time kill assay

#### Time kill assay of butanolic extract and purified compound of *Penicillium* sp.

**Figure 2:**
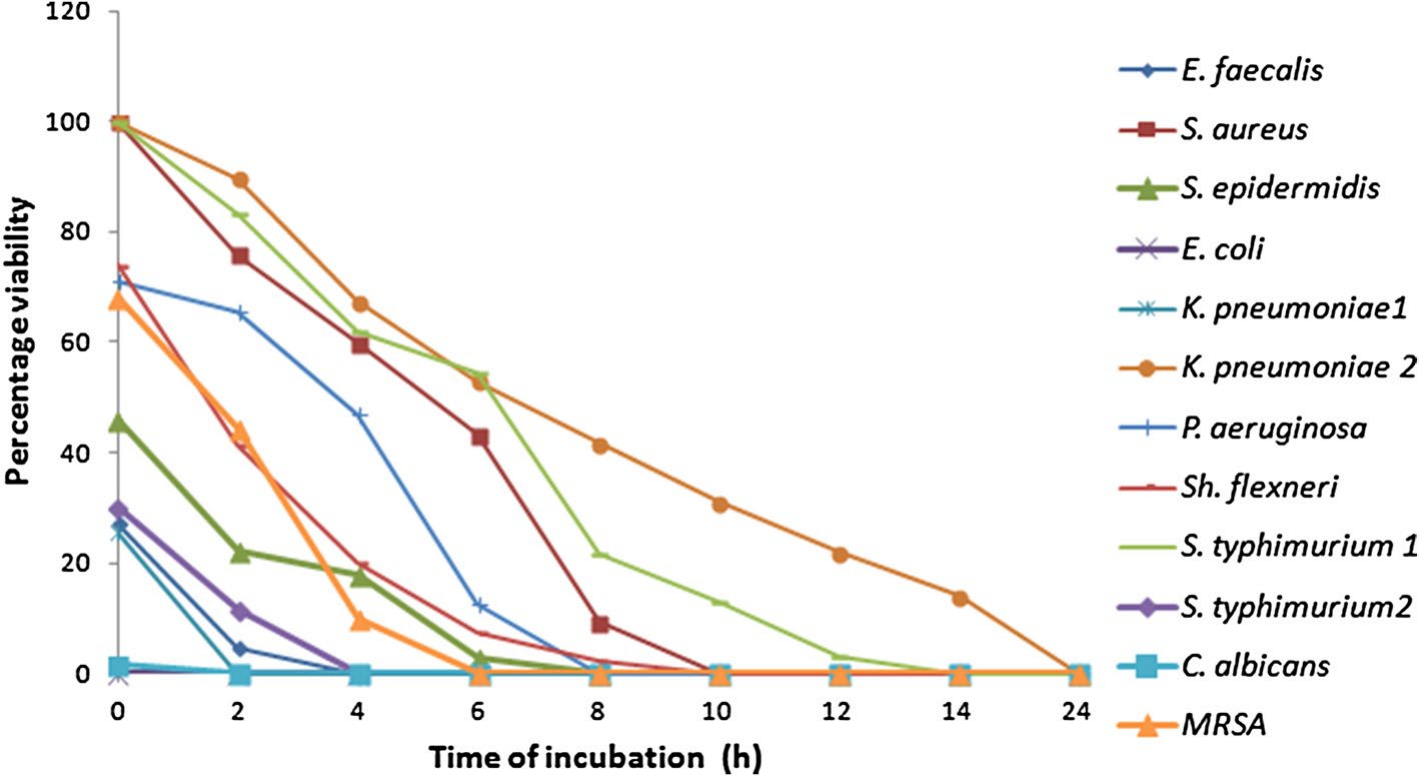
Time kill studies of butanolic extract of *Penicillium* sp.

On the basis of 1× MIC, viable cell count studies were carried out. A stock solution 25 mg/mL of butanolic extract of *Penicillium* sp. and 10 mg/mL for purified compound respectively was prepared. Complete killing of *E. coli* was observed at 0 h of incubation. *K. pneumoniae* 1 and *C. albicans* got completely killed at 2 h. *E. faecalis* took 4 h for complete killing, MRSA 6 h, *S. epidermidis* and *P. aeruginosa* got completely killed at 8 h whereas *S. aureus* and *Sh.flexneri* were killed at 10 h of incubation. *S. typhimurium* 1 took 14 h for complete inhibition and *K. pneumoniae2* took the longest time of 24 h for complete killing (Figure 2).

**Figure 3:**
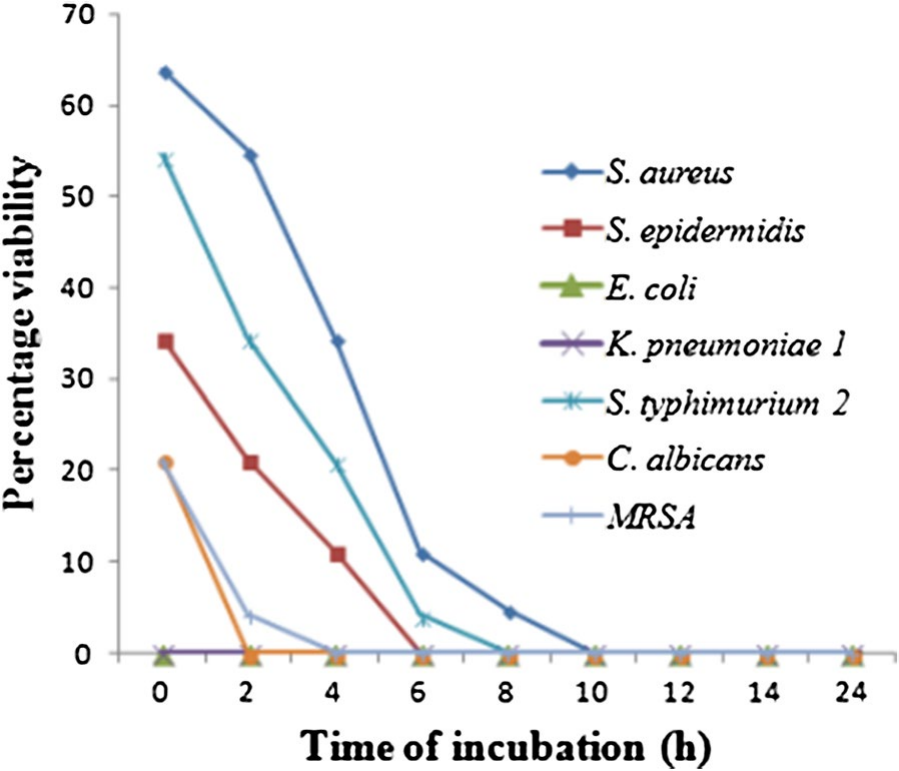
Time kill studies of purified compound.

Time kill assay using purified compound was also studied by viable cell count method. Among the tested organism, complete killing of *E. coli* and *K. pneumoniae* 1 was observed at 0 h. *C. albicans* got killed at 2 h of incubation, whereas *MRSA* and *S. typhimurium* 2 took 4 h whereas *S. epidermidis* took 6 h of incubation. *S. aureus* took the longest time and killed at 10 h of incubation (Figure 3).

### Post antibiotic effect (PAE)

**Figure 4:**
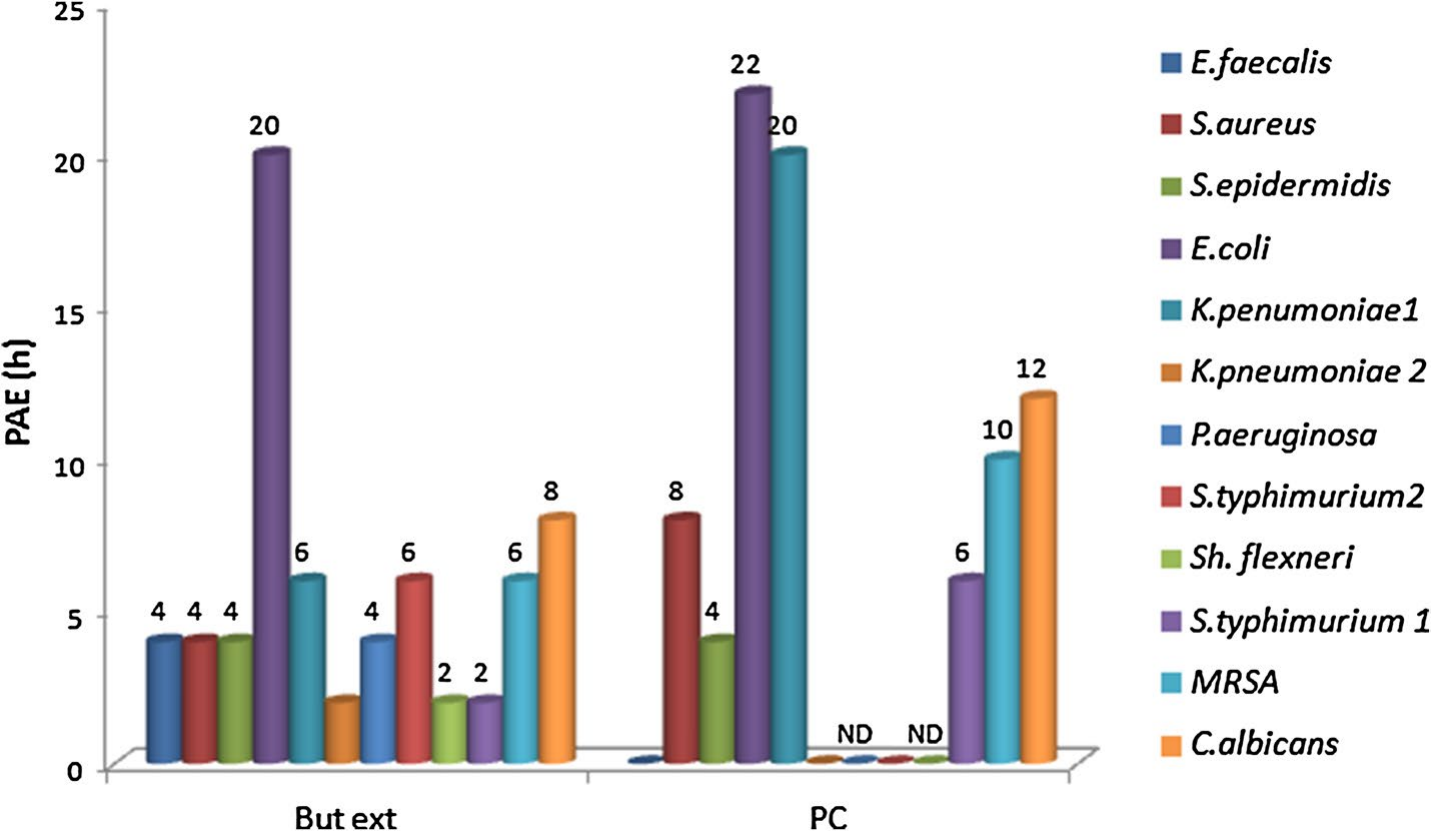
Post antibiotic effect of the butanolic extract and pure compound.

PAE is persistent suppression of bacterial growth after their brief exposure (1 or 2 h) to an antimicrobial agent even in the absence of host defense mechanisms. The concentrations used in kill time assay were applied in PAE studies. Butanolic extract of *Penicillium* sp. and the purified compound induced a varied PAE amongst test organisms and was concentration dependent. Butanolic extract of *Penicillium* sp. induce PAE ranging from 2 to 20 h in the microorganisms tested. *Sh. flexneri* 2 h, *S. aureus* 4 h, *S. epidermidis* 4 h, MRSA 6 h, *K. pneumoniae* 1 6 h, *S. typhimurium* 2 6 h, *C. albicans* 8 h and *E. coli* 20 h, Similarly, purified compound induced PAE ranging from 4 h to 22 h. *S. epidermidis* 4 h, *S*. *typhimurium* 2 6 h, *S. aureus* 8 h, MRSA 10 h, *C. albicans* 12 h, *K. pneumoniae* 1 20 h, and *E. coli* 22 h (Figure 4).

### Toxicity testing

To work out the biosafety of the butanolic extract and purified compound they were subjected to Ames mutagenicity test and MTT cytotoxicity assay. While performing the Ames test the number of revertant colonies in the positive control was numerous, whereas the bacteria incubated with the butanolic extract and the purified compound did not show any revertant colonies. The glucose minimal agar media plates layered with top agar containing excess of biotin and no histidine also served as control as no colonies were observed.

In MTT assay, since the reduction of 3-[(4, 5-dimethylthiazol-2-yl)-2, 5-diphenyl] tetrazolium bromide (MTT) can only occur in metabolically active cells, as the mitochondrial succinate dehydrogenase enzymes in living cells reduce the yellow colored water soluble substrate MTT into an insoluble, formazan crystals which are then dissolved in DMSO and the absorbance of purple colored solutions directly represents the viability of the cells. In the present studies, 90 and 92% of the viable cells were observed in the butanolic extract and the purified compound respectively, showing both to be noncytotoxic.

### Cytotoxic activity against human cancer cell lines

In the present study, purified compound was tested for its cytotoxicity against human cancer cell lines. The compound was found to be endowed with valuable cytotoxic potential against all tested human cancer cell lines (Table 3). The purified compound was found to display promising cytotoxicity with IC_50_ = 28 against leukemia cancer cell line (THP-1). Similarly, against prostate cancer cell line (PC-3), considerable inhibitory activity with (IC_50_ = 31) of the compound was observed. While for breast cancer cell line (MCF-7) the IC_50_ observed was 35 and for lung cancer cell line (A549), +the compound displayed IC_50_ > 50.

**Table 3:** Percentage growth inhibition against different cancer cell lines

Compounds	Conc (µg/mL)	A549	THP-1	PC-3	Colo-205
		Lung	Leukemia	Prostate	Colon
KB14	1	25	0	10	0
	5	28	10	27	20
	10	31	22	38	39
	30	55	39	45	50
	50	75	65	70	72
	IC_50_	28	>50	31	35
5-FU	1	72	74	–	–
Adriamycin	1	67	70	–	80
Mitomycin-c	1	–	67	71	–

### Flow cytometery analysis of nuclear DNA

**Figure 5:**
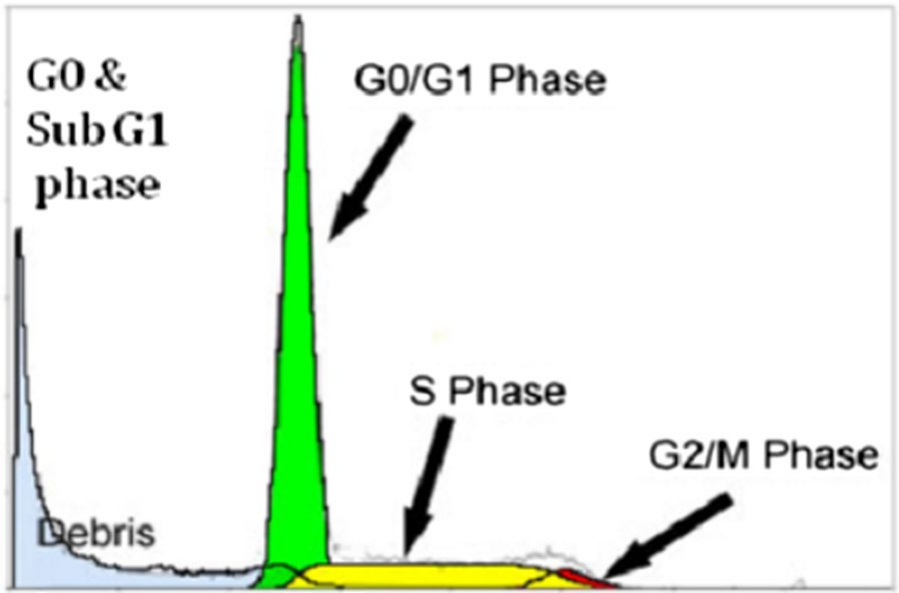
Overview of flowcytometric analysis of nuclear DNA.

**Figure 6:**
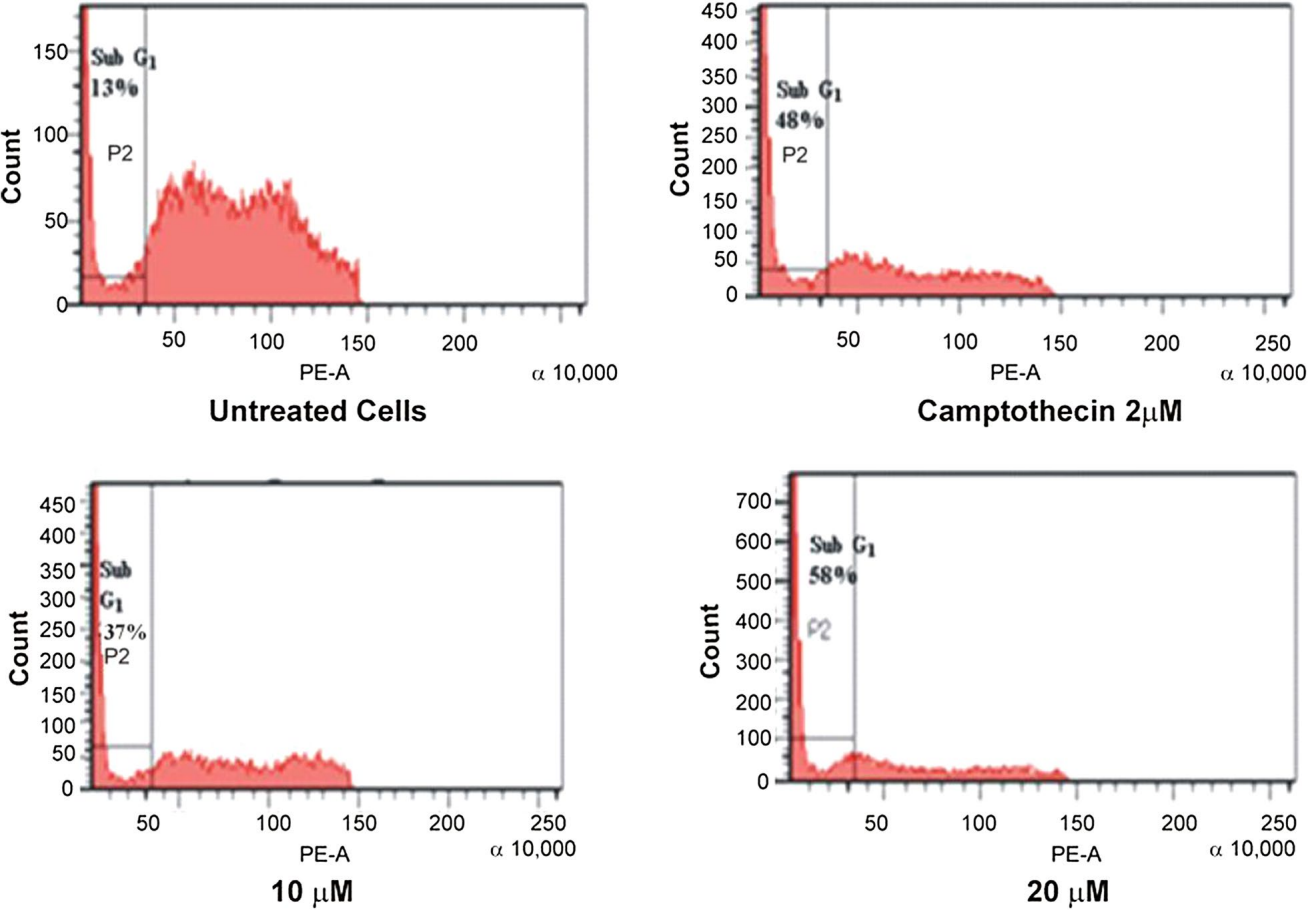
Flow cytometric analysis of nuclear DNA treated compound and the standard.

Morphological change is a primary indication of cell apoptosis. One of the specific characteristics of cell apoptosis is DNA fragmentation, which leads to the appearance of hypodiploid cells. The cell cycle is a key factor in unregulated cell proliferation leading to cancer and differential sensitivity to apoptosis is linked to the distinct phases of cell cycle **(**Pucci et al. 2000). The hypo diploid sub-G1 DNA fraction (<2n DNA) was found to increase from 37 to 58% for compound in a concentration dependent manner (Figures 5, 6). In contrast to camptothecin (positive control) showed 48% hypo diploid DNA content at a concentration of 2 µM.

These results indicate the capability of the purified compound to induce apoptosis in lung cancer (A549) cells.

## Discussion

The emerging problem of increasing resistance led the researchers to look for the novel sources for antimicrobial agents or novel compounds from the existing sources. *Penicillium* spp. have been well known for their bioactive compounds with wide variety of biological activities. A number of *Penicillium* spp. have been studied but a lot more still remain untapped. *Penicillium* sp. in this study has been evaluated for its antimicrobial potential and further the active compound has been purified. Even small changes in the culture medium may not only impact the quantity of certain compounds but also the general metabolic profile of microorganisms. The application of statistical experimental design techniques in the fermentation process development can thus result in improvement of product yield and reduce process variability.

The statistical optimization of various nutritional parameters by RSM resulted in 1.1–1.9 folds enhancement in antimicrobial activity. A good agreement between the predicted and experimental results verified the validity of the model and the improvement of antimicrobial activity indicated the RSM to be a powerful tool for determining the exact optimal values of the individual factors and the maximum response value. Our results corroborate well with other studies where the activity was also enhanced by 1.1–1.8 folds (Wang et al. 2011; Zhengyan et al. 2012).

**Figure 7:**
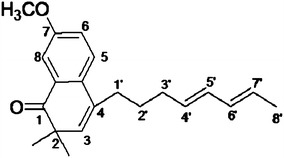
7-Methoxy-2,2-dimethyl-4-octa-4′,6′-dienyl-*2H*-napthalene-1-one.

The purified compound is apparently novel compound with broad spectrum antimicrobial activity. The structure is elucidated on the basis of spectroscopic techniques. Proton NMR of the compound showed a signal of C8-H appeared as singlet at *δ* 9.35 downfield due to anisotropic effect and hydrogen bonding with nearing carbonyl group. The aliphatic multiplet was also observed in the region of *δ* 1.92–1.87 and *δ* 1.40–1.24. The ^13^C NMR spectrum showed aromatic and olefinic carbon resonances in the region of *δ* 169.1–166.4. The signals of aliphatic carbons appeared at the region of *δ* 57.2–15.1 and signal of carbonyl group appeared at *δ* 170.2, which further analyzed by stretching appeared in IR spectrum at 1,666 cm^−1^. The structure of compound was further corroborated by mass spectrum which showed a molecular ion peak at *m*/*z* 333.1766 [M + Na] ^+^. On the basis of these observations the purified compound is proposed to be **7-methoxy-2,2-dimethyl-4-octa-4′,6′-dienyl-2*H*-napthalene-1-one** (Figure 7).

MIC of butanolic extract of the fungi ranged from (0.1–20 mg/mL) which supported the data obtained by agar well diffusion assay. The importance of the study was further highlighted where the purified compound showed better or equal activity when compared with standard antibiotics. MIC of amphotericin B against *C. albicans* was found to be 99 µg/mL whereas, purified compound was effective at much lower MIC of 1 µg/mL. The study showed its further importance when MRSA showed sensitivity to the purified compound with MIC of 5 µg/mL Similarly, compound showed significant inhibitory potential against *K. pneumoniae* 1 *with* MIC 1 µg/mL which is comparable with the gentamicin having MIC of 1 µg/mL. *S. aureus* also showed MIC of 2 µg/mL, *S. epidermidis*, 2 µg/mL. The kill kinetics provides the more accurate description of antimicrobial agents than does the MIC (Mandal et al. 2009). Time kill studies not only give the information about the nature of the antimicrobial agent whether the particular agent is bactericidal or bacteriostatic but also the time taken by the antimicrobial agent for complete killing of the microorganism. Viable cell count studies which showed the effectiveness of the compound with complete killing of *E. coli*, and *K. pneumoniae* 1 at 0 h and our observations are better than previous viable cell count studies with medicinal plants (Kaur and Arora 2009). The viable cell count studies thus endorse that the compound is having potent bactericidal activity.

The design of antimicrobial dosing regimens is mainly based on the susceptibility of pathogen and pharmacokinetic parameters such as drug concentration in serum and tissue. PAE is being applied increasingly to allow antimicrobial dosing regimens to be developed in a more scientific manner. Different antimicrobials induce varied duration of PAE against different types of microbes. The purified compound was found to be effective even after removing it and showed the PAE ranging from 4 to 22 h. More prolonged intermittent dosing regimens would apply primarily to antimicrobials that exhibit a prolonged PAE. On the other hand more continuous dosing would be necessary for antimicrobials that exhibit a shorter PAE or lack of PAE (Lorian 1996).

Further the isolated compound did not show any cytotoxicity as done by MTT assay and mutagenicity by Ames test indicating its suitability to proceed for further pharmacological use.

Many compounds showing biological activities have also been listed in the literature from *Penicillium* sp. having no mutagenicity and cytotoxicity (Kaur et al. 2014; Arora and Chandra 2011). Cancer is a multifaceted and multimechanistic disease, which is one of the leading causes of death. Beside surgical and other interventions, chemotherapy remains the mainstay of treatment of cancer (Harrison et al. 2009). The importance of this study was further highlighted when the compound was found to be endowed with valuable cytotoxic potential against all tested human cancer cell lines. Further the compound induces apoptosis in lung cancer (A549) cells as observed by flow cytometry.

This study conclude that the antimicrobial activity of the purified compound was found to be comparable with standard drugs. Moreover the compound was found to be noncytotoxic which will facilitate the further studies to gain better understanding of the production of bioactive metabolites from fungi. Further, the compound also showed promising anticancerous activity which could be further use for various pharmaceutical purposes.

## **Additional file**

Additional file 1:
**Figure S1.** Mass spectrum of the purified compound.
