# Supplementary material for: Isolation, purification and characterization of novel antimicrobial compound 7-methoxy-2,2-dimethyl-4-octa-4′,6′-dienyl-2H-napthalene-1-one from Penicillium sp. and its cytotoxicity studies
Source: AMB Express. 2015 Jul 4;5:40. doi: 10.1186/s13568-015-0120-9 (PMC4492345; doi:10.1186/s13568-015-0120-9)
Supplement: Additional file 1: — Figure S1. Mass spectrum of the purified compound. [file 13568_2015_120_MOESM1_ESM.pdf]

Isolation, Purification and Characterization of novel Antimicrobial Compound 7-Methoxy-2,2-dimethyl-4-octa-4′,6′-dienyl-2H-napthalene-1-one from *Penicillium* sp. and its cytotoxicity studies.

AMB Express

Harpreet Kaur[a], Jemimah Gesare Onsare[a] , Vishal Sharma[b] and Daljit Singh Arora[a *]

[a] Microbial Technology Laboratory, Department of Microbiology

[b] Department of Pharmaceutical Sciences, Guru Nanak Dev University, Amritsar-143005, India

Tel. No. 91-183-2258802-09 Ext. 3316, Fax No. 91-183-2258819-20

*Corresponding author: Prof. Daljit Singh Arora, Microbial Technology Laboratory

Department of Microbiology, Guru Nanak Dev University, Amritsar-143005, India

E-mail: daljit_02@yahoo.co.in

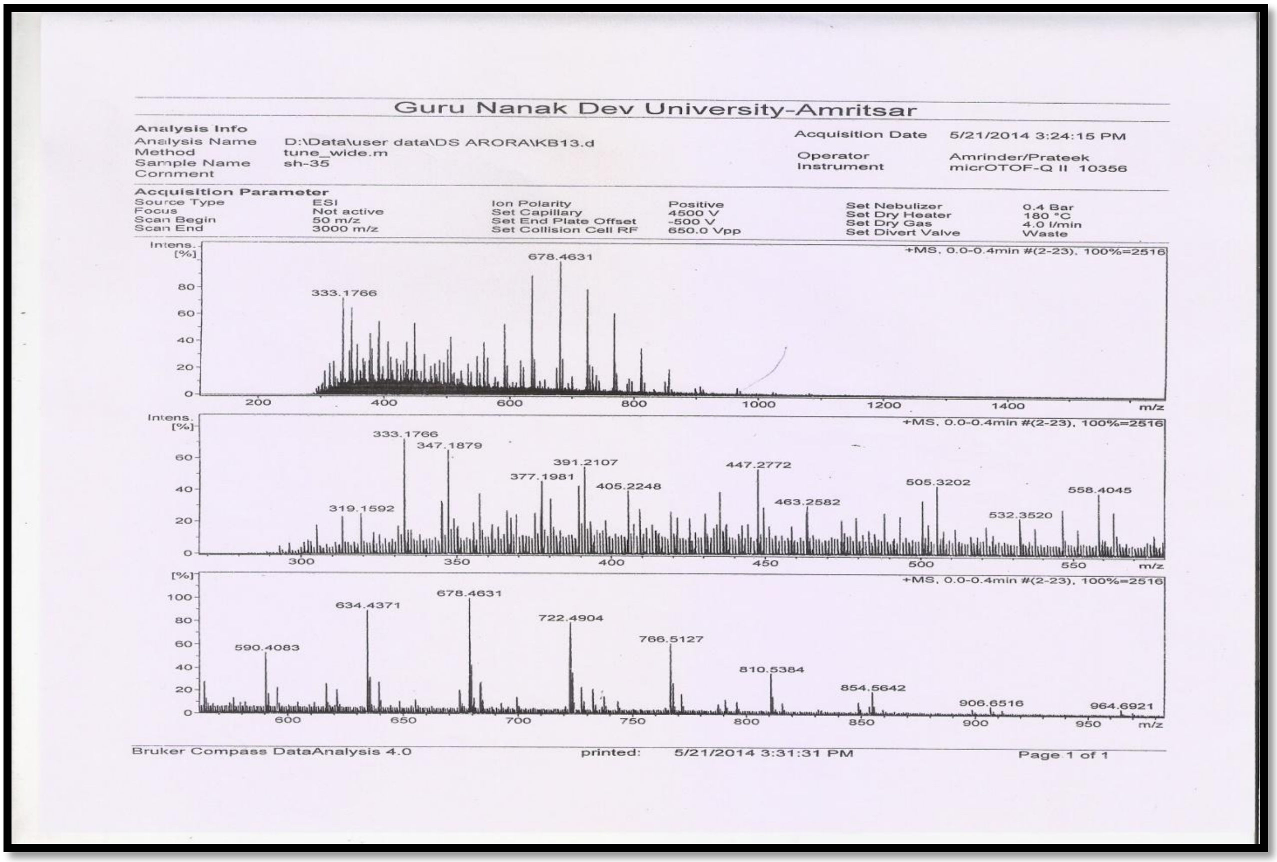

Fig. S1 Mass spectrum of the purified compound
